# Severe Prinzmetal's Angina Inducing Ventricular Fibrillation Cardiac Arrest

**DOI:** 10.1155/2020/3030878

**Published:** 2020-02-07

**Authors:** Bashar Khiatah, David Philips, Jonathan Dukes, Amanda Frugoli

**Affiliations:** ^1^Department of Internal Medicine, Community Memorial Hospital, 147 N Brent St, Ventura, CA 93003, USA; ^2^Cardiology Associates Medical Group, Ventura, CA, USA; ^3^Department of GME Internal Medicine, Community Memorial Hospital, Ventura, CA, USA

## Abstract

Prinzmetal's angina is a vascular spasm of the coronary artery that can mimic acute coronary syndrome. It is rarely responsible for ventricular arrhythmias and cardiac arrest; however, survivors with these complications are at increased risk for recurrent ventricular arrhythmias and sudden cardiac death. This is true despite the presence of normal cardiac function and optimal medical therapy. Thus, this select population should be considered for an implantable cardioverter defibrillator (ICD). In this case vignette, we describe a healthy 48-year-old female with ventricular fibrillation arrest, followed by recurrent ventricular tachyarrhythmias caused by Prinzmetal's angina.

## 1. Introduction

Vasospastic angina (VSA), variant angina (VA), or Prinzmetal's angina is a focal spasm of one or more coronary arteries, without clinically significant atherosclerosis or atherosclerotic plaque. These spasms are responsible for inducing anginal symptoms and temporary ischemia [1, 2]. Research has shown many eliciting factors that increase the sensitivity of the coronary arteries, which in turn cause a hyperconstrictive reaction of the smooth muscles in the coronary arteries [3]. For example, endothelial dysfunction in noncritical plaque can facilitate the vasospasm, which we believe is the electing factor in our case. Rarely, Prinzmetal's angina is responsible for cardiac arrest [4]. Despite optimal medical treatment with calcium channel blockers and nitrates, 5% to 30% of patients continue to have recurrent anginal episodes. Both myocardial infarction and arrhythmia resulting in sudden cardiac death may occur [5]. Thus, treatment choices for this population of patients can be challenging. While vasodilator therapy provides relief of anginal symptoms and ventricular arrhythmia episodes, implantable cardioverter defibrillator (ICD) implantation is the treatment of choice in the case of recurrence, combined with medical therapy [6].

## 2. Clinical Case

A 48-year-old healthy, athletic Japanese female with a past medical history of right renal agenesis presented to the emergency department after being found unconscious by her husband. This occurred just minutes after she reported having severe 10/10 chest pressure with radiation to her jaw. Her husband called 911, and emergency medical service arrived within 7 min. She was found to be in ventricular fibrillation arrest and CPR was immediately started. No epinephrine was administered, but she was defibrillated twice with return of spontaneous circulation. Subsequently, she was brought to the hospital for further evaluation.

The patient states that she has never had this before and has no prior cardiac history. A detailed review of systems included 2-3 loose stools a day, for several days, which started shortly after completing a 30-mile marathon earlier in the week. She reports being unable to finish the run, due to recurrent anginal symptoms. Otherwise, she denied any significant symptoms. Her family history consists of a mother with breast cancer and a father with no significant medical history. Socially, the patient is married and just moved to the United States from Japan 2 years ago. She quit smoking 15 years ago and previously was smoking 6 cigarettes a day for approximately 10 years. She denied any prior recreational drug use. She reports drinking one beer a day.

**Figure 1:**
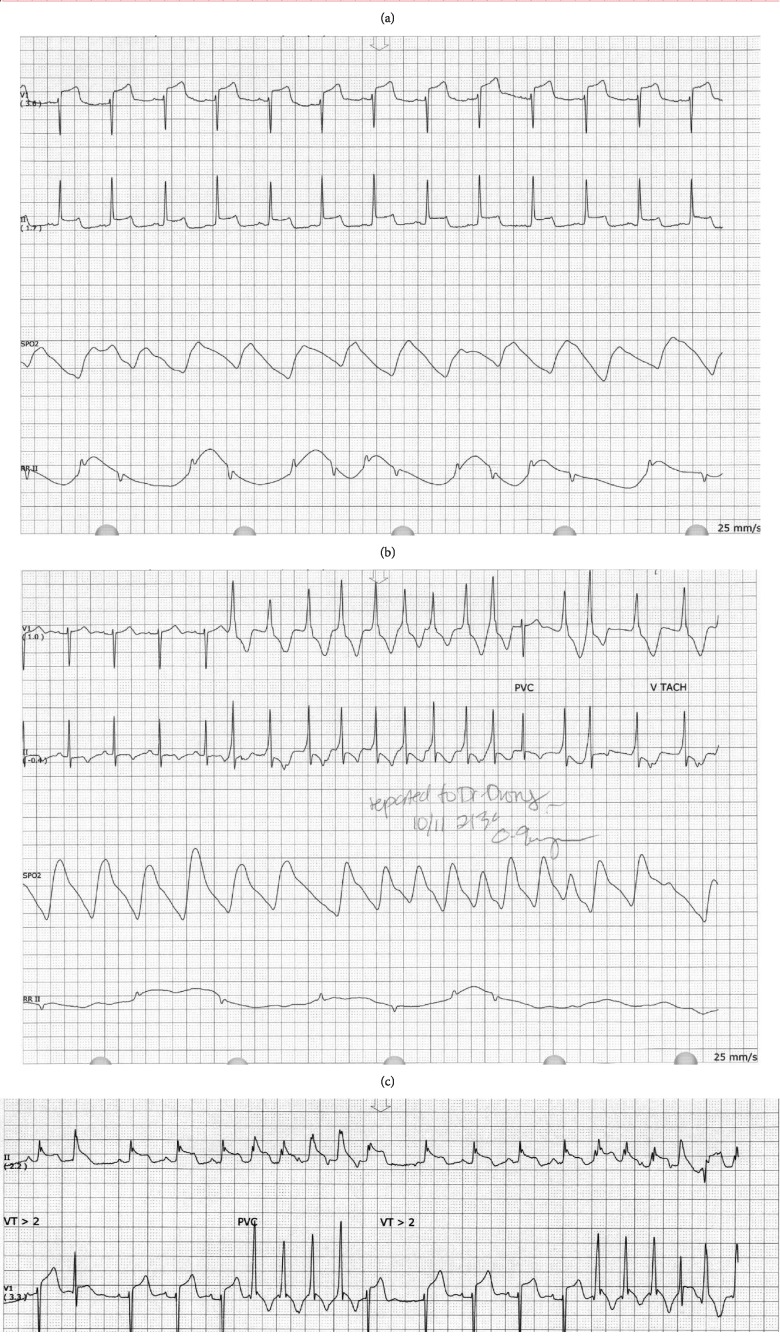
(a) Normal sinus rhythm, no ST segment elevations or depressions, and nonspecific T wave abnormalities. (b) Dynamic ST elevation on ECG. (c, d) Nonsustained ventricular tachycardia.

Her emergency room labs included a normal troponin, as well as hypokalemia at 2.7. Her ECG showed normal sinus rhythm, no ST segment elevations or depressions, and nonspecific T wave abnormalities (Figure 1). She was started on medical therapy with aspirin 325 mg po daily, 80 mEq of potassium, metoprolol 12.5 mg BID, and a heparin drip. Her follow-up studies demonstrated minimally uptrending troponins after 6 hours, with a peak level of 0.18 ng/ml. A transthoracic echocardiogram was completed and was unremarkable, demonstrating preserved left ventricular systolic function and normal wall motion.

**Figure 2:**
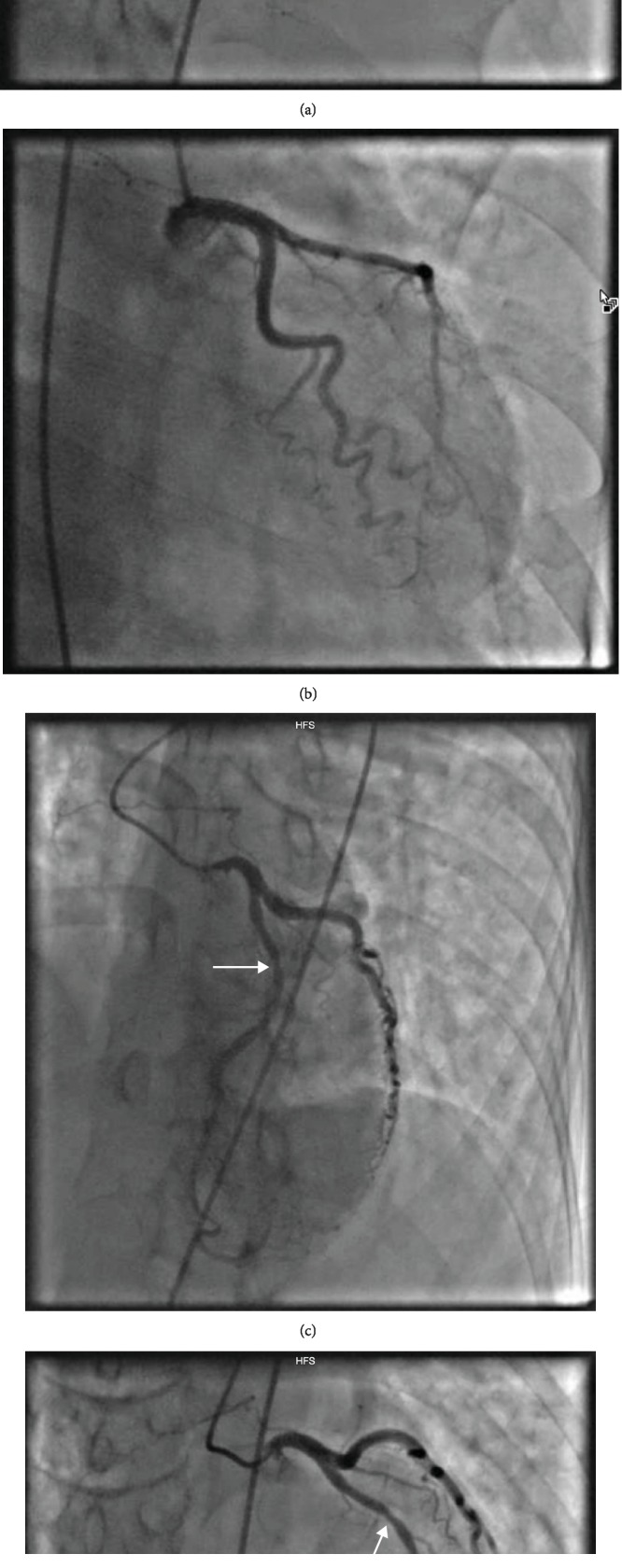
(a) Normal right coronary artery. (b) Normal left main and circumflex artery. (c, d) Left anterior descending artery.

A subsequent cardiac catheterization was performed, which showed no obstructive atherosclerotic disease. She did have mild mid left anterior descending artery stenosis, which did improve with intracoronary nitroglycerin (Figure 2). Otherwise, her coronary arteries were normal.

Her clinical course within the first 24 hours of admission was complicated by recurrent episodes of chest pain, associated with dynamic ST elevation on ECG (Figure 1(b)). She subsequently developed nonsustained ventricular tachycardia (Figures 1(c) and 1(d)). Metoprolol was discontinued, and she was started on amlodipine 5 mg and isosorbide mononitrate 30 mg daily. An external defibrillator LifeVest was ordered.

Additional work-up at this point included a procainamide drug challenge, in order to exclude Brugada syndrome. The patient was given 10 mg/kg of procainamide over 10 min. Her baseline ECG demonstrated normal sinus rhythm, with no notable ST segment abnormalities in the precordial leads V1-V2. No changes in the ST segments or T waves occurred with the procainamide drug challenge. Other possible etiologies for recurrent ventricular arrhythmias were considered but were felt to be less likely the cause in her case: long QT interval (normal QTc on exam and all other ECGs), catecholaminergic polymorphic ventricular tachycardia ((CPVT) VT events were monomorphic and occurred at rest), short QT, and early repolarization (no ECG evidence). Autoimmune etiology was excluded with negative serology including antinuclear antibody, anti-double-stranded deoxyribonucleic acid, and rheumatoid factor. A cardiac magnetic resonance imaging was scheduled to evaluate for infiltrative disease and arrhythmogenic right ventricular cardiomyopathy (ARVC).

Given the presentation of cardiac arrest and her ongoing nonsustained ventricular tachycardia, the tentative plan at this time is to proceed with ICD implantation. After the initiation of the calcium channel blocker and long-acting nitrate, the patient's symptoms and events completely subsided. She was discharged home on these medications and the LifeVest, with a follow-up appointment for ICD placement and magnetic resonance imaging. A couple of weeks after, her MRI was negative for any infiltrative disease and ICD was implanted.

## 3. Discussion

VSA is a widespread diagnosis that includes documented spontaneous attacks of angina pectoris elicited by coronary epicardial vasospasm (EV) and/or coronary microvascular dysfunction (CMD). The diagnostic criteria for VSA, as proposed by the Coronary Vasomotor Disorders International Study Group (COVADIS) [7], includes nitrate sensitive angina with one of the following: rest angina; marked diurnal variation in exercise tolerance; hyperventilation-induced episode or calcium channel blocker-sensitive angina (7); transient ischemic ECG changes, including 0.1 mV ST elevation or depression or new negative U waves in at least two contiguous leads (7); and total or subtotal coronary artery spasm with angina and ischemic ECG changes, either spontaneously or in response to a provocative stimulus (7). Prinzmetal's angina differs from traditional stable angina pectoris anatomically, as it is not driven by atherosclerotic lumen encroachment within the coronary vasculature [8].

Although VSA may coexist with coronary microvascular disorders and/or structural CAD, it is a clinical entity that involves hyperreactivity of the epicardial arteries to vasoconstrictor stimuli [4]. The significance of diagnosing VSA is tied to the major complications correlated with this disorder, including acute myocardial infarction, arrhythmias, or sudden cardiac death (SCD) [9–11], and, in turn, the potential to prevent these life-threatening events by using nitrates and calcium channel blockers and by avoiding potential vasospasm stimuli (2).

Treatment for these patients consists of smoking cessation, weight loss, and avoiding psychological stress or excessive exercise. Also, cardiac rehabilitation and exercise training with behavioral therapy is a cornerstone in therapy [12]. Medical therapies consist of calcium channel blockers and nitrates, with preference given to calcium channel blockers due to concern that long-acting nitrates may produce nitrate intolerance. However, combination therapy with a calcium channel blocker and a nitrate may have a synergistic effect and provide relief when a patient has VSA refractory to monotherapy. Despite the association of vasospasm with atherosclerotic lesions, data regarding the use of aspirin is currently lacking; however, based on current knowledge, low-dose aspirin (<100 mg daily) appears to be safe and may be effective in preventing acute attacks [13]. High-dose aspirin (≥325 mg daily) and beta blockers should both be avoided, as they may elicit an episode of VSA [14]. Finally, invasive treatment includes stent implantation, implantable cardioverter defibrillator, and partial sympathetic denervation. These options are case by case dependent. For example, in a patient with consistent vasospasm due to arterial injury, stenting is a reasonable option. ICD implantation, on the other hand, may be considered for secondary prevention in patients who have had a cardiac arrest secondary to VSA [15]. A recent publication in the *Journal of Arrhythmia*, by Akiko et al., suggested that combined medical treatment using CCB with an ICD may be effective for reducing recurrent ventricular dysrhythmias for some patients with coronary vasospasm who have been successfully resuscitated from ventricular fibrillation or ventricular tachycardia arrest [16]. Additionally, a retrospective review over 18 years by Ahn et al. evaluated a small number of patients with aborted sudden cardiac death (ASCD) and ICD and found there was a nonsignificant trend of cardiac deaths in patients with ICDs compared to those without [17].

Although vasospastic angina rarely presents as ventricular arrhythmias, patients are at higher risk of sudden cardiac death or, in our patient's case, ASCD if they have the following risk factors: hypertension, hyperlipidemia, multivessel spasm, and spasm involving the left anterior descending artery [17]. Recent retrospective review of patients with vasospastic angina with and without aborted sudden cardiac death demonstrated that patients with aborted sudden cardiac death have a higher incidence of cardiac and all-cause mortality [17–19].

In our case, this young healthy female presented with her first attack after starting a marathon, which may have been the inducing factor for her spasm. She would subsequently have recurrent events in the absence of a clear inciting event, including following correction of her hypokalemia, with ECGs suggesting alternating coronary artery involvement not consistent with her very mild single atherosclerotic LAD artery lesion. Her episodic angina and associated nonsustained ventricular tachycardia at rest were resistant to calcium channel blockers alone. She did respond, however, to combination therapy with the addition of a long-acting nitrate and demonstrated stability for discharge. In light of her initial presentation and these recurring events, she was determined to be at high risk for recurrent sudden cardiac death. Arrangements for a LifeVest as a bridge to ICD placement was coordinated.

VSA is an uncommon diagnosis but should be considered for atypical presentations of angina and ECG changes that are not attributed to obstructive coronary artery disease or unstable plaque rupture. This is true for patients, such as ours, in which the presentation is also associated with documented ventricular dysrhythmias and sudden cardiac death. As a diagnosis of exclusion, evaluation for other causes needs to be completed. Pharmaceutical treatment also needs to be individualized and tailored. Additionally, many reports have highlighted the difficulty in predicting the risk of recurrent events. Given the potentially high mortality associated with these arrhythmias, ICD implantation in these patients should be considered. The decision for ICD implantation in patients who experience a significant ventricular arrhythmia should be individualized, taking into consideration known and potentially reversible risk factors for future adverse events, as well as the patient's preferences.
